# Fluticasone/formoterol combination therapy is as effective as fluticasone/salmeterol in the treatment of asthma, but has a more rapid onset of action: an open-label, randomized study

**DOI:** 10.1186/1471-2466-11-28

**Published:** 2011-05-23

**Authors:** Anna Bodzenta-Lukaszyk, Andrzej Dymek, Kirsten McAulay, Heikki Mansikka

**Affiliations:** 1 Department of Allergology and Internal Medicine, Medical University of Białystok, Białystok, Poland; 2 Medical Center Lucyna Andrzej Dymek, Strzelce Opolskie, Poland; 3 Mundipharma Research Limited, Cambridge, UK

## Abstract

**Background:**

The inhaled corticosteroid (ICS) fluticasone propionate (fluticasone) and the long-acting β_2_-agonist (LABA) formoterol fumarate (formoterol) are being made available as a combination product (fluticasone/formoterol, ***flutiform***^®^) in a single aerosol inhaler. This 12-week, open-label, randomized, active-controlled, parallel-group, multicentre, phase 3 study compared the efficacy and safety of fluticasone/formoterol with the commercially available combination product fluticasone/salmeterol.

**Methods:**

Patients aged ≥ 18 years (N = 202) with mild-to-moderate–severe, persistent asthma for ≥ 6 months prior to screening were included in the study. After a screening phase (4–10 days), eligible patients were randomized 1:1 to receive fluticasone/formoterol or fluticasone/salmeterol during the 12-week treatment period. The primary objective was to demonstrate non-inferiority of fluticasone/formoterol versus fluticasone/salmeterol, measured by pre-dose forced expiratory volume in the first second (FEV_1_), at week 12.

**Results:**

Fluticasone/formoterol was comparable to fluticasone/salmeterol for the primary efficacy endpoint, mean pre-dose FEV_1 _at week 12. The new combination was also comparable to fluticasone/salmeterol for change from baseline to week 12 in pre-dose FEV_1_, change from pre-dose FEV_1 _at baseline to 2-hour post-dose FEV_1 _at week 12 and discontinuations due to lack of efficacy. Importantly, fluticasone/formoterol was superior to fluticasone/salmeterol in time to onset of action throughout the duration of the study. The two treatments demonstrated similar results for various other secondary efficacy parameters, including other lung function tests, patient-reported outcomes, rescue medication use, asthma exacerbations and Asthma Quality of Life Questionnaire scores. Fluticasone/formoterol was well tolerated and had a good safety profile that was similar to fluticasone/salmeterol.

**Conclusions:**

The results of this study indicate that fluticasone/formoterol is as effective as fluticasone/salmeterol, and has a more rapid onset of action, reflecting the faster bronchodilatory effects of formoterol compared with those of salmeterol. If patients perceive the benefits of therapy with fluticasone/formoterol more rapidly than with fluticasone/salmeterol, this could have a positive impact on preference and adherence.

**Trial Registration:**

ClinicalTrials.gov: NCT00476073

## Background

Asthma is one of the most common chronic diseases, affecting an estimated 300 million people worldwide. Its prevalence continues to rise in parallel with the increasing urbanization of communities around the world, and an estimated 100 million more people may be affected by 2025 [1]. At an individual level, asthma can have a considerable impact on the quality of life of both patients with asthma and their caregivers [2]. The vast economic burden of asthma comprises both direct costs, such as emergency care, hospitalizations and medications, and indirect costs, largely driven by absenteeism and reduced productivity [3]. In many regions, asthma-associated mortality has declined in recent years, in line with improved management strategies. Despite this, it is estimated that asthma still accounts for approximately 1 in 250 deaths worldwide [1].

Recent data from Europe suggest that over 50% of patients have asthma that is not well controlled [4]. This is largely due to suboptimal use of their medication [5]. It has been suggested that inadequate levels of asthma control account for over half of the existing economic cost of the disease [6,7]. Furthermore, patient and physician perceptions of treatment effectiveness in practice may be inaccurate. The International Asthma Patient Insight Research (INSPIRE) study revealed that 87% of patients with asthma that was not well controlled classed their asthma control as relatively good [8]. Coupled with inaccuracies in physicians' assessments of their patients' asthma control levels [9], these misconceptions probably contribute to the poor adherence of patients to asthma therapy [10].

If adherence to asthma therapy regimens is to be improved, it is important to consider not only the efficacy of treatment but also patients' acceptance of it. There is evidence to suggest that concurrent administration of inhaled corticosteroids (ICSs) and long-acting β_2_-agonists (LABAs) results in a synergistic interaction [11]. In addition, use of only a single inhaler to administer both drugs is likely to improve patient adherence compared with regimens involving treatments administered separately [11,12]. While the benefits of ICS/LABA combination therapies are well established, it is also important to consider the specific components of the combination in order to optimize levels of patient acceptance. The anti-inflammatory effects of the ICS fluticasone propionate (fluticasone) are rapid and sustained [13,14]. Fluticasone is available in a single inhaler with salmeterol (fluticasone/salmeterol), which has proven clinical effectiveness [15]. However, formoterol fumarate (formoterol), as the most rapidly acting LABA (similar time to onset of action to salbutamol [16]), is the bronchodilator of choice for more recently available combination products with budesonide and beclometasone.

Formoterol is now being made available with fluticasone in a single aerosol inhaler (fluticasone/formoterol, ***flutiform***^®^) for the maintenance treatment of asthma in patients whose symptoms are not adequately controlled with an ICS plus an 'as-needed' inhaled short-acting β_2_-agonist, as well as in patients whose asthma is already controlled with both an ICS and a LABA. This phase 3 clinical study compared the efficacy and safety of fluticasone/formoterol with fluticasone/salmeterol in adult patients with mild-to-moderate–severe, persistent asthma.

## Methods

### Study design, setting and objectives

This was an open-label, randomized, active-controlled, parallel-group, phase 3 study, conducted at 25 centres across five European countries (Germany, Hungary, Poland, Romania and the UK; clinicaltrials.gov identifier: NCT00476073). The study was performed in accordance with the Declaration of Helsinki and Good Clinical Practice guidelines, and approved by independent ethics committees in each of the participating countries (Germany, Ethik-Kommission der Landesärztekammer Rheinland-Pfalz; Hungary, Egészségügyi Tudományos Tanács Klinikai Farmakológiai Etikai Bizottsága; Poland, Ethics Committee Medical University in Bialystok; Romania, Academia de Stiinte Medicale, Comisia Nationala de Etica pentru Studiul Clinic al Medicamentului; UK, North West Research Ethics Committee). Written informed consent was obtained from all patients. The study was initiated (first patient, first visit) on 23 April 2007 and was completed (last patient, last visit) on 13 March 2008.

The study was designed to demonstrate non-inferiority of fluticasone/formoterol compared with fluticasone/salmeterol in controlling mild-to-moderate–severe persistent asthma in adult patients. The primary objective of the study was to demonstrate non-inferiority of fluticasone/formoterol to fluticasone/salmeterol based on mean pre-dose forced expiratory volume in the first second (FEV_1_). Secondary objectives of the study were to compare the two treatments with regards to: discontinuations due to lack of efficacy; time to onset of action (defined as 'the first time point post-dose at which the FEV_1 _value was ≥ 12% increased, compared with pre-dose'); peak expiratory flow rates (PEFRs) and other lung function parameters; amount of rescue medication use; asthma symptom scores; sleep disturbance due to asthma; daily oral or parenteral corticosteroid doses; asthma exacerbations; patient assessments of study medication; Asthma Quality of Life Questionnaire (AQLQ) scores; and spontaneously reported adverse events (AEs). The study included a screening phase of 4–10 days to evaluate eligibility for participation. On completion of the screening phase, patients were reassessed for eligibility and randomized to receive study treatment.

### Inclusion and exclusion criteria

Men and women (aged ≥ 18 years) with mild-to-moderate–severe, persistent asthma for ≥ 6 months prior to screening were included in the study. Patients were required to demonstrate a FEV_1 _of ≥ 40% and ≤ 85% of predicted normal values [17] during the screening phase following appropriate withholding of asthma medications (if applicable). Patients were also required to show reversibility of ≥ 15% in FEV_1 _after salbutamol inhalation (two actuations, 100 μg per actuation) in order to be eligible for randomization. Only patients who could demonstrate correct inhaler technique were entered into the study.

Among the exclusion criteria were: life-threatening asthma within the past year; hospitalization or emergency department visit for asthma in the 4 weeks prior to screening; systemic corticosteroid use in the month prior to screening; omalizumab use in the past 6 months; use of a leukotriene receptor antagonist in the week before screening; a smoking history that was either recent (in the 12 months prior to screening) or equivalent to ≥ 10 pack-years (e.g. at least 20 cigarettes/day for 10 years); significant non-reversible active pulmonary disease; and clinically significant respiratory tract infection in the 4 weeks prior to screening. Also prohibited was recent use (in the past week) of β-blocking agents, tricyclic antidepressants, monoamine oxidase inhibitors, astemizole, quinidine-type anti-arrhythmics or potent CYP3A4 inhibitors. Current use of medications that would have an effect on bronchospasm and/or lung function was also a criterion for exclusion.

### Interventions and randomization

Patients were randomized 1:1 to one of two treatment groups, with two dose options available within each group. Eligible patients were assigned a unique randomization number selected sequentially from a randomization list via an interactive voice randomization system. A random permuted block design was used to obtain a 1:1 ratio. Patients randomized to receive fluticasone/formoterol were to take two actuations of 50/5 μg or 125/5 μg every 12 hours (i.e. 100/10 μg or 250/10 μg twice daily). Patients randomized to receive fluticasone/salmeterol were to take two actuations of 50/25 μg or 125/25 μg every 12 hours (i.e. 100/50 μg or 250/50 μg twice daily). Both study treatments were administered via a hydrofluoroalkane pressurized metered-dose inhaler with an AeroChamber^® ^Plus spacer device (GlaxoSmithKline, Uxbridge, UK).

The starting dose of each medication was selected based on each patient's asthma history and pre-study asthma medication. Patients who required ICSs at a dose of 100–250 μg/day fluticasone or equivalent received the low dose of study medication, while patients who required > 250 μg/day (up to 1000 μg/day) fluticasone or equivalent received the high dose of study medication. Patients receiving the low dose of study medication were permitted to switch to the high dose during the treatment period if their asthma was not controlled, at the investigator's discretion. The use of salbutamol (two actuations, 100 μg per actuation, up to four occasions per day) was permitted as rescue medication.

### Compliance

At weeks 2, 6 and 12 the approximate number of actuations made since the previous visit was recorded. Compliance was calculated as the number of actuations taken (i.e. sum of values from weeks 2, 6 and 12) as a percentage of the number of actuations that should have been taken (i.e. number of study days multiplied by four).

### Prior and concomitant medication

All concomitant medications and therapies that were ongoing at the date of informed consent were recorded.

### Efficacy assessments

During the 12-week treatment phase, patients underwent lung function tests (forced vital capacity [FVC], maximum expiratory flow at 25%, 50% and 75% of volume to exhale [MEF_25_, MEF_50_, MEF_75_] and PEFR) at weeks 0 (baseline), 2, 6 and 12. Each of these tests was performed in the 30 minutes prior to administration of study medication and repeated 5, 10, 30, 60, 90 and 120 minutes post-treatment. PEFR measurements were assessed for reproducibility by completing a minimum of three acceptable manoeuvres (based on American Thoracic Society/European Respiratory Society guidelines) with the two largest FEV_1 _measurements not varying by more than 0.15 L. The measurement with the best FEV_1 _was selected for recording of PEFR. In addition, patients recorded their morning and evening peak flow rates daily. Patients were also assessed for asthma exacerbations (at weeks 2, 6 and 12), completed the AQLQ [18] (at weeks 0 and 12) and performed an assessment of their study medication at week 12 (5-point scale ranging from very poor to very good). Mild-to-moderate exacerbations were: pre-dose morning PEFR > 30% below baseline on ≥ 2 consecutive days; night awakening due to asthma ≥ 2 consecutive days; or use of salbutamol rescue medication > 4 times per day for ≥ 2 consecutive days. Severe exacerbations were deterioration in asthma requiring additional therapy, or emergency visit or hospitalization due to asthma. On a daily basis throughout the treatment phase, patients recorded the following information by means of an electronic diary: rescue medication use; asthma symptom scores; sleep disturbance due to asthma; morning and evening PEFR; and study medication taken.

### Safety assessments

Safety assessments were made at weeks 2, 6 and 12 after the start of treatment. Adverse events were documented based on spontaneous reporting, patient interview and diary entries. Clinical laboratory tests (haematology, biochemistry and urinalysis) were performed at screening, week 6 and week 12. Vital signs (blood pressure, heart rate, respiration rate and oral temperature) were assessed at screening and week 12. Twelve-lead electrocardiograms were recorded at screening, week 6 and week 12. Patients were followed up by telephone 14 days after completion of/discontinuation from the study to report any ongoing or new AEs.

### Sample size

The sample size was focused on the difference in the 12-week FEV_1 _values analysed within a linear model with the baseline FEV_1 _value as a covariate. It was assumed that the observed treatment difference would not exceed 0.02 and the standard deviation would be 0.5. The non-inferiority bound was fixed to 0.2, corresponding to an effect size of 0.4, which could be interpreted as 'mild-to-moderate'. For a two-sided level of significance (α = 0.05, power of 80%), 113 patients per treatment group were required. Assuming a correlation between the 12-week and baseline FEV_1 _values of approximately 0.5, the sample size would be reduced to 85 patients per treatment group. The comparison was focused on the per protocol (PP) population. Assuming that approximately 10% of the randomized patients would not be part of the PP population, 200 patients needed to be randomized to this study.

### Analysis populations

The relevant study sets were as follows: safety population, all patients who received study treatment and had at least one post-dose safety assessment; full analysis set (FAS), all randomized patients who received study treatment and had at least one post-dose primary efficacy (FEV_1_) measurement; PP population, all FAS patients who completed the study without major protocol violations affecting the primary efficacy (FEV_1_) endpoint.

### Statistical analyses

The primary endpoint (pre-dose FEV_1 _at week 12) and several secondary efficacy endpoints (change from baseline to week 12 in pre-dose FEV_1_, change from pre-dose FEV_1 _at baseline to 2-hour post-dose FEV_1 _at week 12, discontinuations due to lack of efficacy and time to onset of action) were tested on the PP population in a confirmatory manner, with pre-defined non-inferiority limits. Non-inferiority for the primary endpoint would be concluded if the lower limit of the 95% confidence interval (CI) was ≥ –0.2 L. For discontinuation due to lack of efficacy, the upper limit of the 95% CI was required to be ≤ 10% (for the difference between the percentage of patients in each treatment group) for non-inferiority to be demonstrated.

Non-inferiority of fluticasone/formoterol to fluticasone/salmeterol for the primary endpoint was tested on the PP population using an analysis of covariance (ANCOVA) with treatment and dose group as factors and the pre-dose FEV_1 _values at week 0 as a linear covariate, and centre as a random effect. Secondary endpoints were analysed as follows: change in pre- and post-dose FEV_1_, PEFR measurements, other lung function parameters and AQLQ scores were analysed using ANCOVA; the difference in percentages and 95% CIs were calculated for discontinuations due to lack of efficacy; time to onset of action was analysed using the multiple failures time model [19]; rescue medication use was analysed using a Wilcoxon rank sum test; asthma symptoms and sleep disturbance scores were analysed using a linear model; patient assessment of asthma medication was analysed using a proportional odds model; asthma exacerbations were analysed using Fisher's exact test. Other endpoints, including safety parameters, were summarized using descriptive statistics. All hypothesis tests were two-sided and conducted at the 5% error level.

## Results

### Recruitment and patient flow

**Figure 1:**
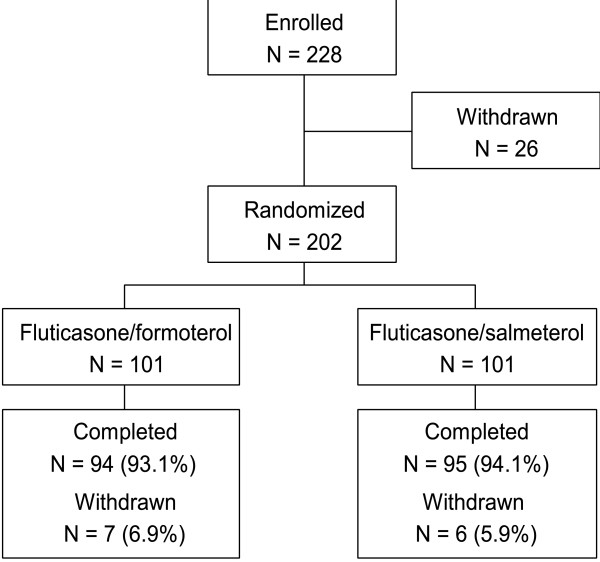
**Participant flow**.

The study was initiated (first patient, first visit) on 23 April 2007 and was completed (last patient, last visit) on 13 March 2008. Of the 202 patients randomized (101 per treatment group), 189 (93.6%) completed the study and 13 (6.4%) discontinued prematurely (Figure 1). Of the 13 patients who did not complete the study, five (2.5%) withdrew by choice (four in the fluticasone/formoterol group, one in the fluticasone/salmeterol group), four (2.0%) were withdrawn due to lack of therapeutic effect (two in each group), three (1.5%) were withdrawn for administrative reasons (all in the fluticasone/salmeterol group), and one (0.5%) was withdrawn due to an AE (fluticasone/formoterol group). The safety population, PP population and FAS comprised 202, 191 and 202 patients, respectively.

### Baseline demographics and characteristics

The demographics and asthma characteristics of patients in the fluticasone/formoterol and fluticasone/salmeterol groups were similar (Table 1). There were no relevant differences between the treatment groups. All patients demonstrated an FEV_1 _of ≥ 40% to ≤ 85% of predicted normal values, as described in the inclusion criteria. FEV_1 _reversibility ranged from 15% to 82% across the two groups. Mean baseline AQLQ scores were 4.9 ± 1.0 in the fluticasone/formoterol group and 4.7 ± 1.0 in the fluticasone/salmeterol group. Inhaled corticosteroid and LABA use was similar in the two groups, as was ICS dose (based on the Global Initiative for Asthma guideline on equipotency of ICS [20]).

**Table 1:** Demographic characteristics, asthma status and treatment at screening

	Fluticasone/formoterol	Fluticasone/salmeterol
	
	(N = 101)	(N = 101)
**Age (years)**		

Mean ± SD	47.6 ± 12.6	46.0 ± 12.9

Median (range)	50.0 (18–75)	47.0 (18–76)

**Gender**		

Male, n (%)	47 (46.5)	39 (38.6)

Female, n (%)	54 (53.5)	62 (61.4)

**Race**		

Caucasian, n (%)	101 (100.0)	101 (100.0)

**Weight (kg), mean ± SD**	79.0 ± 15.3	76.1 ± 16.3

**Height (cm), mean ± SD**	170.2 ± 9.9	167.6 ± 8.4

**BMI (kg/m**^**2**^**), mean ± SD**	27.3 ± 4.8	27.1 ± 5.3

**FEV**_**1 **_**pre-salbutamol (L), mean ± SD**	2.11 ± 0.56	2.11 ± 0.52

**FEV**_**1 **_**post-salbutamol (L), mean ± SD**	2.70 ± 0.79	2.63 ± 0.66

**Predicted FEV**_**1 **_**(L), mean ± SD**	3.20 ± 0.73	3.08 ± 0.65

**FEV**_**1 **_**% predicted, mean ± SD (range)**	66.1 ± 10.1 (41–85)	68.6 ± 9.2 (44–85)

**FEV**_**1 **_**reversibility (%), mean ± SD**	27.6 ± 12.8	24.9 ± 9.9

**Treatment**		
ICS, n (%)	93 (92.1)	94 (93.1)
ICS dose (μg), median (range)*	500 (100–1000)	400 (100–1000)
LABA, n (%)	78 (77.2)	78 (77.2)

BMI, body mass index; FEV_1_, forced expiratory volume in the first second; SD, standard deviation; ICS, inhaled corticosteroid; LABA, long-acting β_2_-agonist.

*Based on fluticasone equivalent, according to the GINA guideline on equipotency of ICS [20].

### Starting dose of study medication

A total of 73 (72.3%) patients in the fluticasone/formoterol group and 76 (75.2%) patients in the fluticasone/salmeterol group started the study on the high-dose option. Only a very small number of patients required a change in dose strength (to a higher dose) during the study (five in the fluticasone/formoterol group, three in the fluticasone/salmeterol group).

### Compliance

Compliance was over 75% in 99% of patients in the fluticasone/formoterol group and in 98% of patients in the fluticasone/salmeterol group.

### Primary outcome measure

**Figure 2:**
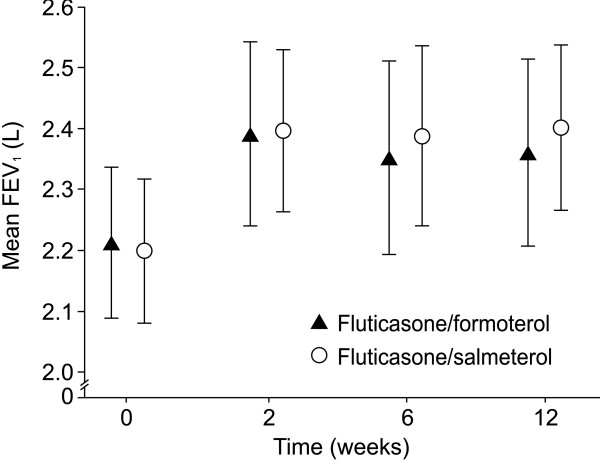
**Mean (± 95% CI) pre-dose FEV**_**1 **_**throughout treatment**. Data shown for per protocol population. Least-squares mean of the treatment difference at week 12: –0.061 L; 95% CI: –0.161, 0.040; *p *= 0.007, demonstrating non-inferiority. CI, confidence interval; FEV_1_, forced expiratory volume in the first second.

In terms of the primary efficacy endpoint, mean pre-dose FEV_1 _at week 12, fluticasone/formoterol was comparable to fluticasone/salmeterol (least-squares [LS] mean of the treatment difference at week 12: –0.061 L; 95% CI: –0.161, 0.040; *p *= 0.007, demonstrating non-inferiority; PP population; Figure 2).

### Secondary outcome measures

With regards to the secondary efficacy endpoints, fluticasone/formoterol was comparable to fluticasone/salmeterol in terms of the change in pre-dose FEV_1 _from baseline to week 12 (LS mean of the treatment difference: –0.061 L; 95% CI: –0.161, 0.040; *p *= 0.007; PP population, Table 2). A supportive analysis, in the form of an ANCOVA that included a treatment by dose group (i.e. higher or lower dose) interaction, further confirmed non-inferiority of fluticasone/formoterol for this secondary parameter. Fluticasone/formoterol was also comparable to fluticasone/salmeterol in terms of the change from pre-dose FEV_1 _at baseline to 2-hour post-dose FEV_1 _at week 12 (LS mean of the treatment difference: –0.013 L; 95% CI: –0.129, 0.103; *p *= 0.002; PP population, Table 2).

**Table 2:** Change in FEV_1 _assessments from baseline to week 12

Parameter	Change from baseline (L) LS mean (95% CI)	Difference between groups (L) LS mean (95% CI)	*p *value for non-inferiority
			
***Pre-dose FEV***_***1***_			
*Fluticasone/formoterol*	0.196 (0.117, 0.275)	–0.061 (–0.161, 0.040)	0.007
		
*Fluticasone/salmeterol*	0.257 (0.177, 0.336)		

***Post-dose FEV***_***1***_			

*Fluticasone/formoterol*	0.464 (0.374, 0.555)	–0.013 (–0.129, 0.103)	0.002
		
*Fluticasone/salmeterol*	0.477 (0.384, 0.569)		

FEV_1_, forced expiratory volume in the first second; LS, least-squares mean.

In the PP population, fluticasone/formoterol was comparable to fluticasone/salmeterol in terms of discontinuation due to lack of efficacy, which occurred once in the fluticasone/formoterol group and twice in the fluticasone/salmeterol group. This translated to a difference of –1.1% (95% CI: –4.6, 2.5), with non-inferiority of fluticasone/formoterol to fluticasone/salmeterol duly demonstrated for the PP population (because upper limit of the CI was ≤ 10%).

#### Time to onset of action

**Figure 3:**
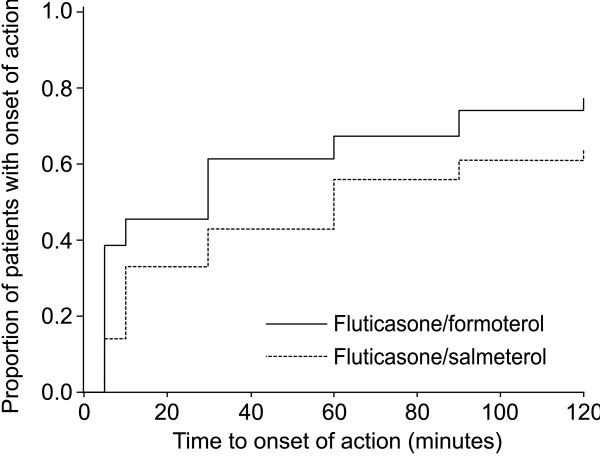
**Time to onset of action of study medication at baseline (day 0; similar plots were obtained at weeks 2, 6 and 12)**. Data based on Kaplan-Meier survival analysis (full analysis set). Onset of action was defined as the first time point post-dose at which FEV_1 _was at least 12% greater than the pre-dose value. Analysis of time to onset of action using the multiple failures time model showed superiority of fluticasone/formoterol over fluticasone/salmeterol (hazard ratio: 1.64; 95% CI: 1.28, 2.10; *p *< 0.001). On day 0, more than twice as many patients had a bronchodilatory response that met the onset of action criterion in the fluticasone/formoterol group than in the fluticasone/salmeterol group at 5 minutes post-dose (39% versus 14%, respectively). In addition, a larger proportion of patients in the fluticasone/formoterol group met the onset of action criteria within 2 hours (post-dose), than in the fluticasone/salmeterol group (77% versus 64%, respectively; day 0). This trend continued over the 12-week treatment period, with consistently more patients achieving the predefined onset of action response in the fluticasone/formoterol group than in the fluticasone/salmeterol group at each post-dose time point (5–120 minutes). CI, confidence interval; FEV_1_, forced expiratory volume in the first second.

Fluticasone/formoterol was superior to fluticasone/salmeterol with regards to the time to onset of action throughout the 12 weeks of the study. Analysis of time to onset of action using the multiple failures time model showed superiority of fluticasone/formoterol over fluticasone/salmeterol (hazard ratio: 1.64; 95% CI: 1.28, 2.10; *p *< 0.001; FAS; see Figure 3 as an example of this effect). The proportion of patients in both treatment groups showing onset of action (onset of action was defined as the first time point post-dose at which FEV_1 _was at least 12% greater than the pre-dose value) at weeks 2, 6 and 12 was lower than at week 0 (baseline), but fluticasone/formoterol continued to have a faster onset of action than fluticasone/salmeterol at all visits.

#### Other secondary outcome measures

Fluticasone/formoterol demonstrated similar efficacy to fluticasone/salmeterol for other secondary parameters, as summarized in Table 3. Mean (range) asthma symptom scores in the first week of the study were 0.74 (0–3.29) and 0.58 (0–3.00) in the fluticasone/formoterol and fluticasone/salmeterol groups, respectively. Corresponding values in the last week of the study were 0.51 (0–3.14) and 0.39 (0–3.43). Changes from baseline in various lung function tests are given in Table 4.

**Table 3:** Summary of treatment differences in secondary efficacy endpoints

Secondary endpoint	Analysis population	Statistical analysis	Difference Fluticasone/formoterol - fluticasone/salmeterol
Discontinuations due to lack of efficacy (%)	PP	%	–1.1
		95% CI^a,b^	–4.6, 2.5*

Time to onset of action of study medication (minutes)	FAS	Hazard ratio	1.64
		95% CI	1.28, 2.10
		*p *value	< 0.001*

Rescue medication use (% of study days)	FAS	HL estimate	2.24
		95% CI	–0.03, 7.06
		*p *value	0.157

Rescue medication use (number of uses)	FAS	HL estimate	0.02
		95% CI	0.00, 0.11
		*p *value	0.164

Pre-dose PEFR at week 12 (L/min)	FAS	LS mean	–13.6
		95% CI	–34.9, 7.6
		*p *value	0.208

2-hour post-dose PEFR at week 12 (L/min)	FAS	LS mean	–10.0
		95% CI	–33.6, 13.7
		*p *value	0.408

Other lung function parameters (FVC, MEF_25_, MEF_50 _and MEF_75_)	FAS	LS mean	No statistically significant differences between the treatment groups
		95% CI	
		*p *value	

Asthma symptom scores (mean values)	FAS	Mean^c^	0.15
		95% CI	–0.04, 0.34
		*p *value	0.122

Sleep disturbance scores (mean values)	FAS	Mean^c^	0.00
		95% CI	–0.12, 0.11
		*p *value	0.975

Asthma exacerbations: mild/moderate	FAS	n (%)	Fluticasone/formoterol: 11 (10.9)
			Fluticasone/salmeterol: 12 (11.9)
		*p *value	> 0.999

Asthma exacerbations: severe	FAS	n (%)	Fluticasone/formoterol: 3 (3.0)
			Fluticasone/salmeterol: 1 (1.0)
		*p *value	0.621

Patient assessment of study medication (scores at week 12)	FAS	Odds ratio	0.495
		95% CI^c^	0.289, 0.848

AQLQ (scores at week 12)	FAS	Mean (SD)	Fluticasone/formoterol: 5.4 (1.1)
			Fluticasone/salmeterol: 5.5 (0.9)
		*p *value	0.051

*Statistically significant non-inferiority (discontinuations due to lack of efficacy) or superiority (time to onset of action) of fluticasone/formoterol versus fluticasone/salmeterol based on sequential gatekeeping approach.

^a^Non-inferiority of fluticasone/formoterol to fluticasone/salmeterol is shown if the upper limit of the 95% CI is ≤ 10%.

^b^*p *value not calculated.

^c^Adjusted mean from linear model on mean score with treatment group as factor.

AQLQ, Asthma Quality of Life Questionnaire; CI, confidence interval; FAS, full analysis set; FVC, forced vital capacity; HL, Hodges Lehmann; LS, least-squares; MEF_25_; MEF_50_; MEF_75_, maximum expiratory flow rate at, respectively, 25%; 50%; and 75% of the volume to exhale; PEFR, peak expiratory flow rate; PP, per protocol population; SD, standard deviation.

**Table 4:** Change in lung function parameters from pre-dose baseline to 2-h post-dose at week 12 in the full analysis set

Parameter	Change from baseline, LS mean (95% CI)	
	
	*Fluticasone/formoterol*	*Fluticasone/salmeterol*
FVC (L)	0.402 (0.296, 0.508)	0.354 (0.247, 0.461)

MEF_25 _(%)	15.3 (10.5, 20.2)	17.3 (12.3, 22.3)

MEF_50 _(%)	43.0 (33.1, 53.0)	51.7 (41.5, 62.0)

MEF_75 _(%)	87.8 (71.9, 103.6)	93.5 (77.5, 109.5)

FVC, forced vital capacity; LS, least-squares; MEF_25_; MEF_50_; MEF_75_, maximum expiratory flow rate at, respectively, 25%; 50%; and 75% of the volume to exhale.

The percentage of patients assessing each medication as good or very good was high in both groups (84% in the fluticasone/formoterol group, 91% in the fluticasone/salmeterol group).

### Safety parameters

The two treatment groups had similar safety and tolerability profiles. The overall rate of AEs was 23.8% in both treatment groups. The majority of AEs were of mild or moderate intensity; severe AEs were experienced by two patients in the fluticasone/formoterol group (2.0%). The most common AEs in both groups were 'infections and infestations', occurring in 13.9% of patients in the fluticasone/formoterol group and 12.9% of patients in the fluticasone/salmeterol group. AEs judged to be related to treatment were documented for one patient in each treatment group. Serious AEs (SAEs) were also reported for one patient in each treatment group. The SAEs experienced by the patient in the fluticasone/formoterol group (haemorrhagic stroke and cardiac arrest, approximately 2 months after randomization) led to withdrawal from the study, and had a fatal outcome. The SAE reported in the fluticasone/salmeterol group was pneumococcal pneumonia. All SAEs were considered unrelated to treatment with study medication by both the investigators and the sponsor.

There were no noteworthy changes in laboratory parameters, vital signs or electrocardiograms. No systemic effects of LABAs were observed (e.g. elevation of serum glucose or reduced serum potassium concentrations).

## Discussion

The efficacy of inhaled fluticasone and of inhaled formoterol is documented extensively in the literature, including as part of ICS/LABA combination therapies with other components [15,21-26]. Prescribing data indicate that fluticasone and formoterol are already co-prescribed and used concurrently in patients with asthma. Now these two components are brought together into a single combination therapy option. It has previously been demonstrated that fluticasone/formoterol has comparable efficacy to its individual components administered concurrently [27].

This study demonstrated that fluticasone/formoterol is comparable to standard fluticasone/salmeterol therapy in terms of the primary endpoint (pre-dose FEV_1 _at week 12), as well as for several secondary efficacy endpoints (change from baseline to week 12 in pre-dose FEV_1_, change from pre-dose FEV_1 _at baseline to 2-hour post-dose FEV_1 _at week 12 and discontinuation due to lack of efficacy). Fluticasone/formoterol also demonstrated similar efficacy to fluticasone/salmeterol for various other measures of lung function, rescue medication use, patient reported outcomes and asthma exacerbations. Furthermore, the safety and tolerability profiles of the two treatments were similar.

Importantly, fluticasone/formoterol was superior to fluticasone/salmeterol in terms of time to onset of action. This is most likely to be due to the rapid onset of action of formoterol, which is equivalent to that of salbutamol [16]. Although the proportion of patients in both treatment groups showing onset of action at weeks 2, 6 and 12 was lower than at week 0 (baseline), reflecting the fact that the patients were not as well controlled at baseline and thus more responsive to study medication, the superiority of fluticasone/formoterol over fluticasone/salmeterol was maintained. The benefit of fluticasone/formoterol in terms of speed of onset of action may be clinically important, given that a consensus panel of practising physicians has suggested that a rapid onset of action might be expected to have a positive impact on the adherence of patients to their treatment [28]. However, it should also be considered that patients who are well controlled may not experience the benefit offered by a medication with a rapid onset of action.

A potential limitation of any randomized clinical trial is the extent to which it reflects real-life practice, particularly bearing in mind the discrepancies between treatment efficacy in controlled trials and the levels of asthma control observed in clinical practice. Clearly, randomized, controlled trials have an important role, but more pragmatic assessments of 'real-world effectiveness' may complement these data, particularly given the relevance of patient preference and adherence in this field [29]. For example, the odds ratio for the overall patient assessment of study medication for fluticasone/formoterol compared with fluticasone/salmeterol was in favour of the latter. Nevertheless, the study medication was assessed as good or very good by 84% of patients in the fluticasone/formoterol group and 91% of those in the fluticasone/salmeterol group. This needs to be considered in the context of a tightly regulated trial setting with good levels of compliance. In real-life practice, where patients adopt self-management strategies and often do not take their medication according to the prescribed regimens, factors such as the speed at which relief is perceived by the patient could have more impact [28,30]. Trials aimed at assessing how different treatments influence individual patients' decision-making need to be carefully designed in order to ensure their relevance to practice [31].

In theory, patients may have been overtreated at baseline and during the study, which could mask any differences between the two treatments. However, lung function parameters, AQLQ scores and asthma symptom scores improved (at least numerically) from baseline in both groups (AQLQ data not shown) suggesting that patients were not overtreated. Nevertheless, in hindsight it may have been useful to have used a standardized asthma control questionnaire throughout the study to provide insights into control at baseline.

It is worth noting that this study was open-label in design. It was not deemed likely, however, that the non-blinded nature of this study had a detrimental effect on the results, because the primary efficacy measure was a physical rather than a subjective endpoint and analysis of the data was blinded until its completion.

## Conclusions

In conclusion, this study has demonstrated that fluticasone/formoterol has comparable efficacy to fluticasone/salmeterol for patients with mild-to-moderate–severe, persistent asthma, with regards to mean pre-dose FEV_1 _at week 12, change from baseline to week 12 in pre-dose FEV_1_, change from pre-dose FEV_1 _at baseline to post-dose FEV_1 _at week 12 and discontinuation due to lack of efficacy. Superiority of fluticasone/formoterol over fluticasone/salmeterol was shown for time to onset of action of study medication over the 12 weeks of the study. Analysis of additional efficacy parameters such as other lung function tests, patient-reported outcomes, rescue medication use, asthma exacerbations and AQLQ scores yielded comparable results for the two treatment groups. Furthermore, fluticasone/formoterol was well tolerated with a good safety profile.

These data suggest that the fluticasone/formoterol combination will offer patients a treatment option that is as effective as fluticasone/salmeterol, but with a more rapid onset of action. There is therefore potential for the new combination to have a positive impact on adherence to treatment, and hence on asthma outcomes.

## Competing interests

AB-L has received speaker fees from AstraZeneca, HAL Allergy, Novartis, Nycomed and Torrex Chiesi. AD declares no competing interests. At the time of study completion, KM and HM were employees of Mundipharma Research Limited, which funded the study and is financing the article-processing charge for this manuscript.

## Authors' contributions

AB-L was the coordinating investigator for this study. AD was a study investigator. KM was the sponsor's study manager. HM was the sponsor's medical officer. All authors have read and approved the final manuscript.

## Pre-publication history

The pre-publication history for this paper can be accessed here:

http://www.biomedcentral.com/1471-2466/11/28/prepub
